# Protective Potential of Antioxidant Enzymes as Vaccines for Schistosomiasis in a Non-Human Primate Model

**DOI:** 10.3389/fimmu.2015.00273

**Published:** 2015-06-02

**Authors:** Claudia Carvalho-Queiroz, Ruth Nyakundi, Paul Ogongo, Hitler Rikoi, Nejat K. Egilmez, Idle O. Farah, Thomas M. Kariuki, Philip T. LoVerde

**Affiliations:** ^1^Department of Biochemistry, University of Texas Health Science Center, San Antonio, TX, USA; ^2^Department of Pathology, University of Texas Health Science Center, San Antonio, TX, USA; ^3^Institute of Primate Research, National Museums of Kenya, Nairobi, Kenya; ^4^Department of Microbiology and Immunology, School of Medicine and Biomedical Sciences, University at Buffalo, Buffalo, NY, USA

**Keywords:** *Schistosoma mansoni*, antioxidants, vaccine, anti-fecundity, baboon

## Abstract

Schistosomiasis remains a major cause of morbidity in the world. The challenge today is not so much in the clinical management of individual patients, but rather in population-based control of transmission in endemic areas. Despite recent large-scale efforts, such as integrated control programs aimed at limiting schistosomiasis by improving education and sanitation, molluscicide treatment programs and chemotherapy with praziquantel, there has only been limited success. There is an urgent need for complementary approaches, such as vaccines. We demonstrated previously that anti-oxidant enzymes, such as Cu–Zn superoxide dismutase (SOD) and glutathione S peroxidase (GPX), when administered as DNA-based vaccines induced significant levels of protection in inbred mice, greater than the target 40% reduction in worm burden compared to controls set as a minimum by the WHO. These results led us to investigate if immunization of non-human primates with antioxidants would stimulate an immune response that could confer protection as a prelude study for human trials. Issues of vaccine toxicity and safety that were difficult to address in mice were also investigated. All baboons in the study were examined clinically throughout the study and no adverse reactions occurred to the immunization. When our outbred baboons were vaccinated with two different formulations of SOD (SmCT-SOD and SmEC-SOD) or one of GPX (SmGPX), they showed a reduction in worm number to varying degrees, when compared with the control group. More pronounced, vaccinated animals showed decreased bloody diarrhea, days of diarrhea, and egg excretion (transmission), as well as reduction of eggs in the liver tissue and in the large intestine (pathology) compared to controls. Specific IgG antibodies were present in sera after immunizations and 10 weeks after challenge infection compared to controls. Peripheral blood mononuclear cells, mesenteric, and inguinal node cells from vaccinated animals proliferated and produced high levels of cytokines and chemokines in response to crude and recombinant antigens compared with controls. All together, these data demonstrate the potential of antioxidants as a vaccine in a non-human primate model.

## Introduction

Schistosomiasis remains a major cause of morbidity in the world. The disease is endemic in 76 countries of the world where it affects some 200 million people (1). Substantial research has established that school-aged children in endemic areas are at significant risk of serious disease resulting from infection with schistosomes (2, 3). Lack of access to clean water and sanitation and inadequate personal hygiene are important shared risk factors for these infections (4).

Currently, chemotherapy with praziquantel (PZQ) is the preferred treatment for schistosomiasis (5–8). Control programs based on mass chemotherapy are complicated by rapid and frequent re-infection and the difficulties and expense of maintaining these programs over a long term (9–12). There is evidence that schistosomes develop drug resistance against PZQ in some regions (13, 14), and there is additional evidence for serious rebound morbidity if regular and periodic treatments are interrupted (15–17). Despite recent large-scale efforts, integrated control programs aimed at limiting schistosomiasis by improving education and sanitation, molluscicide treatment programs to reduce the population of the intermediate snail host, and chemotherapy have had limited success (18). Effective control of schistosomiasis could prevent up to 130,000 deaths and avert up to 25 million disability adjusted life years lost annually (19). The challenge today is not so much in the clinical management of individual patients, but rather in population-based control of transmission (and consequently, of morbidity) in endemic areas. Because current control strategies employing chemotherapy with PZQ have not reduced transmission and morbidity to acceptable levels, there is an urgent need for complementary approaches, such as vaccines for schistosomiasis (20, 21).

Our own studies have focused on antioxidant enzymes as vaccine candidates [recently reviewed by Huang et al. (22)]. We hypothesized that antioxidants play a role in protecting the adult worms from damage derived from reactive oxygen species (23–26). To begin to test this hypothesis, we demonstrated that expression of the schistosome antioxidant enzymes [Cu-Zn superoxide dismutase (SOD); glutathione S peroxidase (GPX)] is developmentally regulated such that the lowest levels of gene expression (as measured by transcription) and enzyme specific activity were in the larval stages, the most susceptible to immune killing, and highest in adult worms, the least susceptible to immune elimination (23, 25, 27–29). To provide direct evidence that antioxidant enzymes were important in immune evasion (25) and thus were viable candidate vaccines, we used DNA vaccination strategies to demonstrate the efficacy of DNA constructs encoding either Cu/Zn cytosolic superoxide dismutase (SmCT-SOD), signal peptide-containing SOD (SmSP-SOD, also known as SmEC-SOD), or glutathione peroxidase (SmGPX) to be protective against *Schistosoma mansoni* infection in a murine challenge model. Employing different doses of plasmid cDNA constructs, mice exhibited a significant level of worm burden reduction when challenged with *S. mansoni* cercariae after immunization with SmCT-SOD (54%) and SmGPX (43.4%) from six independent experiments (29).

The WHO has identified several candidate vaccine antigens for independent evaluation, but none fulfilled the required standards in trials with mice. One of the criteria was a worm burden reduction of >40% in a murine challenge model (30). We have demonstrated that both SmCT-SOD and SmGPX were each capable as DNA-based (plasmid or vaccinia virus vehicles) vaccines to consistently induce significant levels of protection in an *S. mansoni* murine-challenge model, greater than the target 40% reduction in worm burden compared to control set as a minimum by the WHO. We also demonstrated in a mouse model that DNA encoding SmCT-SOD as a vaccine is able to significantly reduce worm burden by targeting adult *S. mansoni* worms 21 days and older (31).

The above results with the antioxidant enzymes in a murine model of *S. mansoni* encouraged us to investigate if immunization of non-human primates with antioxidants would stimulate an immune response that would be safe and that could confer protection.

## Materials and Methods

### Animals and screening

Young wild caught Olive Baboons (*Papio cynocephalus anubis*), both males and females aged 4–5 years, were from schistosomiasis non-endemic areas and habituated and quarantined for 90 days in accordance with good animal welfare standards. The baboons were screened for the presence of several infections and parasites (such as malaria, tuberculosis, leishmaniasis, nematodes, etc.) before they were assigned to the study. For assessing the presence of current *Schistosoma* infections, the fecal material was examined for the presence of eggs and the serum from each animal was checked for the presence of antibodies against soluble egg antigens (SEA). Positive animals were excluded from our study or when necessary successfully treated using ivermectin and/or metro-nidazole as appropriate, months before the start of immunization. Detailed hematological tests certified all animals were in excellent health during their quarantine period. The animals were housed isolated at the facilities of the Institute of Primate Research (IPR), Karen, Nairobi, Kenya. Ethical clearance for these studies was obtained from the Institute for Primate Research IACUC (IPR/SRP3/2004) and the University of Texas Health Science Center at San Antonio IACUC (08039x).

### Preparation of the antioxidant vaccines

cDNA containing the entire open reading frames of SmCT-SOD, SmEC-SOD, SmGPX were previously cloned into the eukaryotic expression vectors pcDNAI/Amp (pc) (31, 32) and VR1055 (Vical, San Diego, CA, USA) (33). Each plasmid preparation was then purified by double gradient centrifugation in CsCl_2_, dialyzed against PBS to remove the CsCl_2_, ethanol precipitated, and re-suspended in sterile sucrose (25% in PBS). The last boost consisted of the respective recombinant proteins incorporated into microspheres made of polylactic acid (PLA). Only recombinant proteins were incorporated into PLA microspheres, as previously described by us (32). Of note, the following terms are used interchangeably throughout the study: SmCT-SOD or CTSOD; SmEC-SOD or ECSOD; SmGPX or GPX; and Vector Only or Control.

### Immunizations and challenge

**Figure 1:**
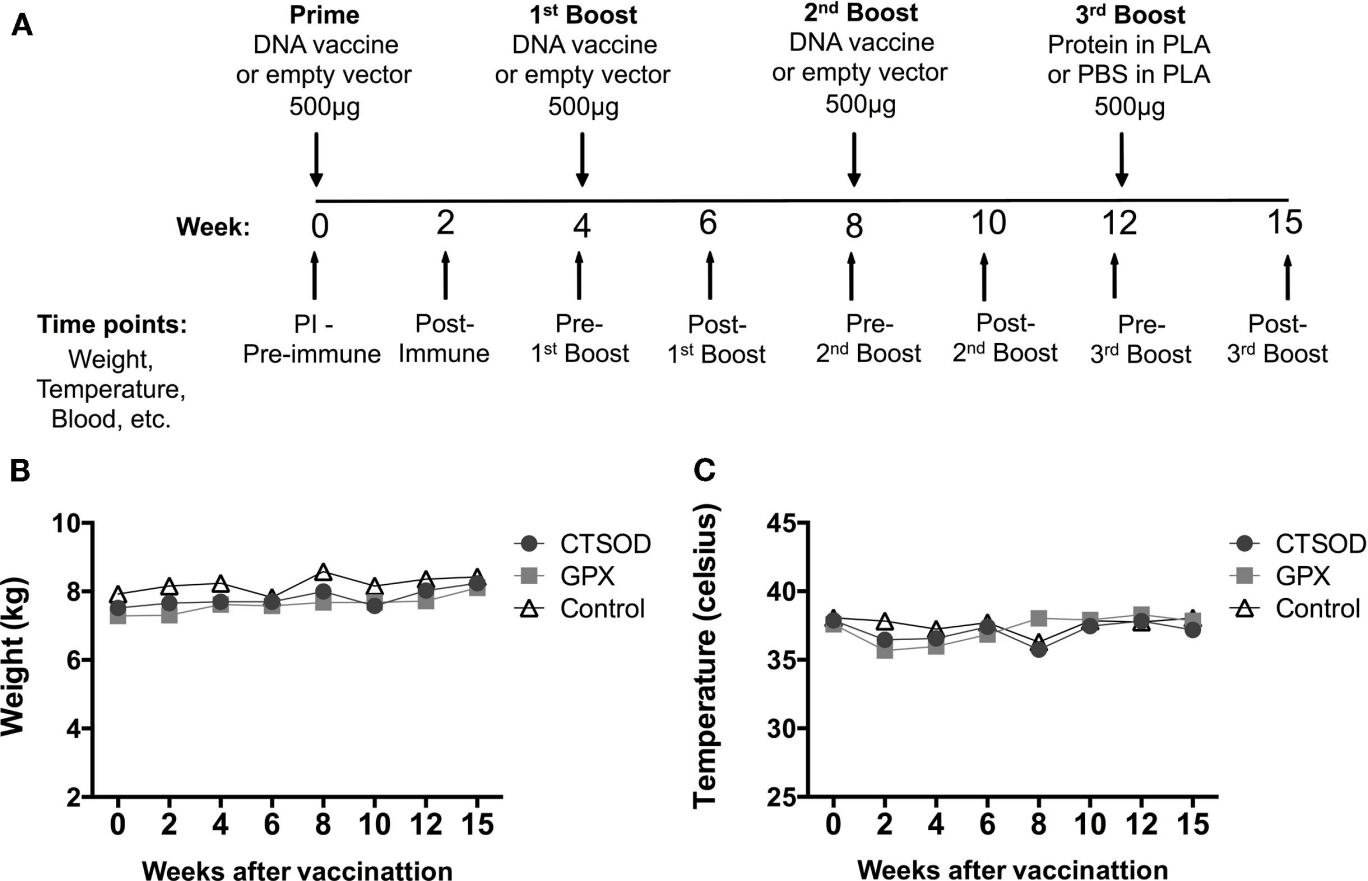
**Experiment 1 scheduling and clinical assessment of safety and toxicity**. In **(A)**, the schematic representation of Experiment 1 protocol where olive baboons (*n* = 5 animals per vaccine group, *n* = 10 controls) were immunized with CTSOD, ECSOD, and GPX DNA-protein based vaccine. For safety and toxicity, the animals were monitored daily for appetite, behavior, and demeanor observations (data not shown) and bi-weekly for weight and temperature. In **(B,C)**, the average results of the bi-weekly monitoring of weight **(B)** and temperature **(C)** from baboons before and after immunizations with antioxidant vaccines during safety and toxicity evaluation. Animals were euthanized at week 26 post-immunization.

**Figure 2:**
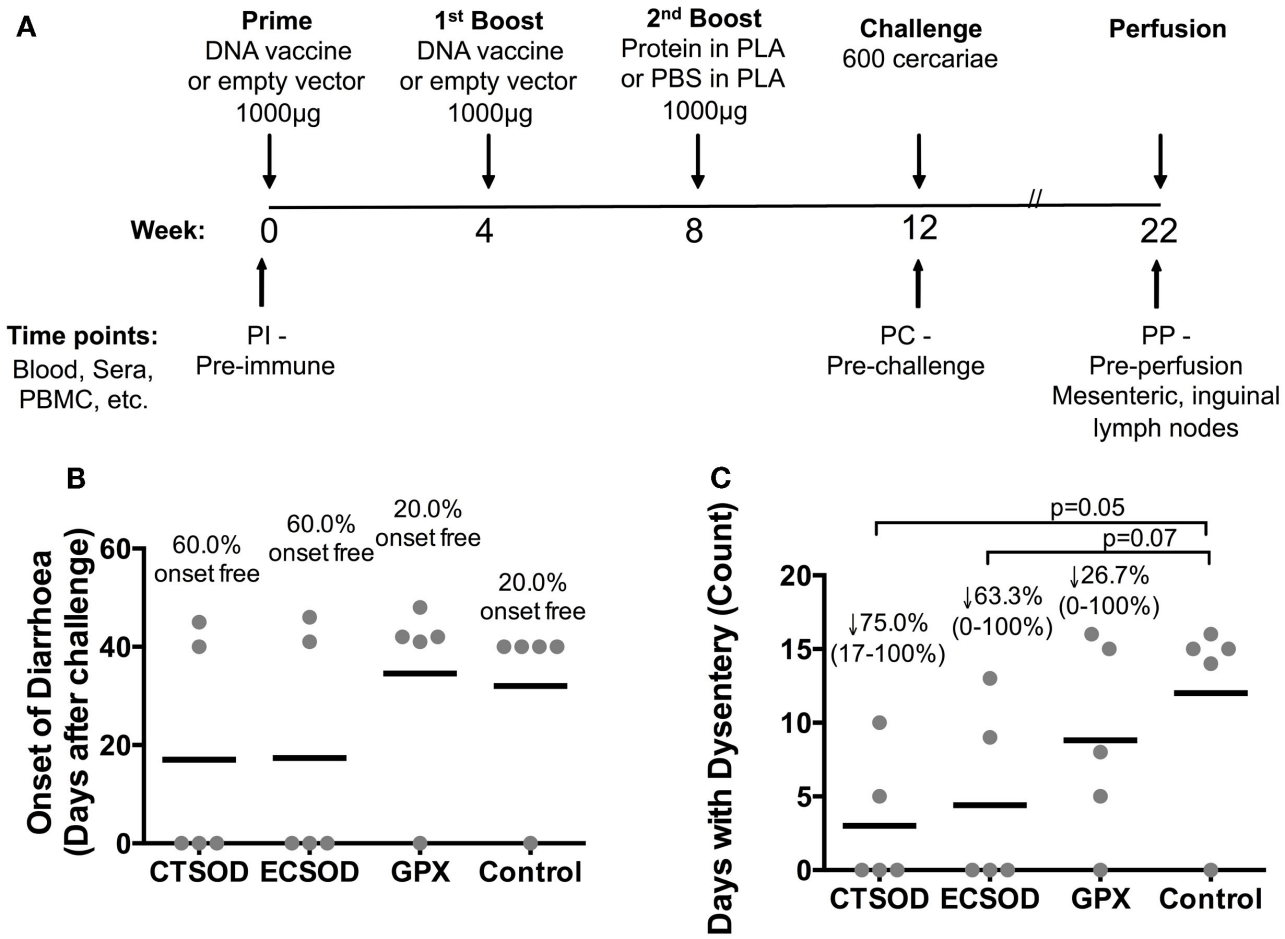
**Experiment 2 scheduling and clinical assessment after challenge infection**. In **(A)**, the schematic representation of the Experiment 2 protocol where olive baboons were immunized with CTSOD, ECSOD, and GPX DNA-protein based vaccine (*n* = 5 animals per each group), and further challenged with 600 *S. mansoni* cercariae. Animals were also assessed for safety and toxicity of vaccination (data not shown). In **(B,C)**, commence of diarrhea and duration of dysentery in days after challenge infection. Individual values (sphere); mean average (dash). The percentage of animals that did not suffer diarrhea is represented in **(B)**. In **(C)**, the percentage of reduction (represented by the arrow), the range (minimal-maximal, represented between brackets), and the statistical *p*-value were expressed in comparison to the mean average of the Control group. Non-parametric (Kruskal–Wallis, Mann–Whitney) tests were applied. Values were considered statistically significant when *p* < 0.05.

Two vaccination experimental protocols were carried out: Experiment 1 (Figure 1A) was for safety and toxicity evaluation after vaccination, while Experiment 2 (Figure 2A) for assessment of the efficacy of vaccination upon challenge with *S. mansoni*. In Experiment 1 (Figure 1A), groups of five olive baboons received 500 μg of purified DNA via two intramuscular (quadriceps) injections with a 26-gage needle in each leg. Each group received a second and third 500 μg dose of their respective DNA, 4 weeks apart. A fourth dose of 500 μg of recombinant proteins encapsulated in PLA microspheres as described (32) was given 4 weeks after the third injection. The control animals were primed and boosted with empty vector DNA, while the last boost consisted of empty PLA microspheres in PBS instead of recombinant proteins.

In Experiment 2 (Figure 2A), groups of five 4- to 5-year-old olive baboons were primed with 1000 μg of purified DNA, boosted once with 1000 μg doses of their respective purified DNA, and once with 1000 μg of the respective recombinant protein encapsulated in PLA microspheres, with all immunizations 4 weeks apart from each other. The control animals were primed and boosted with empty vector DNA, while the last boost consisted of empty PLA microspheres in PBS instead of recombinant proteins. The baboons were then anesthetized using a mixture of xylazine (2% Rompun™) and Ketamine hydrochloride (100 mg/ml; Agrar Holland BV, Soest, The Netherlands) at a dose of 10 mg/kg body weight. After shaving the groin areas of the baboons, 600 cercariae (Kenyan strain) in suspension from individual beakers were poured into a groin pouch and left to penetrate via the skin for 30 min.

### Blood collection for hematology, cell, and serum isolation

All animals were anesthetized with ketamine hydrochloride (10 mg/kg body weight) prior to blood collection. Then, a 10 ml syringe with a 21-gauge needle was used to draw blood from the femoral vein, where it was placed into an EDTA treated tube for hematology (Experiment 1) or used for serum collection (Experiment 2). In addition, 50 ml syringes containing 20 ml of Alsever’s anti-coagulant solution (Sigma) and fitted with a 21-gauge needle were used to draw 20 ml of blood to a total volume of 40 ml blood mixture from the opposite limb (Experiment 2).

In the Experiment 1, as depicted in Figure 1A, the blood was drawn every 2 weeks (with exception of the last time point in which blood was taken 3 weeks after last boost). The eight time points were: week 0 (pre-immune, PI), week 2 (2 weeks post-priming), week 4 (pre-1st boost), week 6 (2 weeks post-1st boost), week 8 (pre-2nd boost), week 10 (2 weeks post-2nd boost), week 12 (pre-3rd boost), and week 15 (3 weeks post-3rd Boost). In the Experiment 2 (Figure 2A), the blood was drawn at three time-points: week 0 (pre-immune, PI), week 12 (pre-challenge, PC), and week 22 (pre-perfusion, PP).

### Clinical assessment for safety and toxicity

The baboons were monitored daily for appetite, behavior, and demeanor, and tested at 2-week intervals for temperature, weight measurements, complete blood counts, and leukocyte differentials at baseline (week 0) and after all immunizations.

### Perfusion

Approximately 10 weeks post-challenge, the baboons were euthanized by intravenous administration of heparinized sodium pentobarbital. For each animal, the viscera were exposed after a median incision. The gross pathology of the liver, intestines, and mesenteric lymph nodes was evaluated. Perfusion of worms from the mesenteric vasculature and the liver was achieved after the administration of citrated saline through the abdominal aorta, as described previously (34), and adult worms were counted. To ensure that all worms were recovered, during the perfusion, the mesenteric veins were manually searched for schistosome worms. The worms were counted, and as an indicator of efficacy, the mean percent protection observed in the experimental groups was determined as the decrease in worm burden of each animal compared to the mean recovery of the control groups (35).

### Parasitological parameters

The weekly assessment of eggs per gram of feces (EPG) from vaccinated and control baboons commencing 6 weeks after *S. mansoni* cercariae challenge was determined by the thick smear Kato-Katz microscope technique (36). For extraction and determination of eggs present in organs, 10% tissue samples (by weight) of liver and intestines were digested overnight in 5% KOH, as described previously (37). The eggs were counted and the percentage of reduction in egg excretion in stool or tissues calculated in relation to the mean average of the control group.

### Enzyme-linked immunosorbent assay

The titers of IgG antibodies in the baboon sera against recombinant antigens derived from *S. mansoni* were determined by ELISA as described with minor modifications (32). Flat-bottom 96-well polystyrene plates (Maxisorp Nunc, GIBCO, Scotland) were coated overnight at 4°C with 50 μl of antigens diluted in PBS 0.15 M, pH 7.2 (PBS) at concentration of 5 μg/ml. After washing (automatic ELISA Washer – MR 5000, Dynatech) and blocking, 50 μl/well of doubling dilution of individual serum or pooled sera diluted in PBS-Tween 20, 0.05% PBS-T were added to each plate and the plates incubated overnight at 4°C. After washing, 50 μl/well of unconjugated goat anti-monkey IgG antibodies (Abd Serotec) were added and the plates were incubated 1 h at 37°C. After the incubation, the plates were washed, and 50 μl of alkaline-phosphatase conjugated donkey anti-goat IgG antibodies (Abd Serotec) were added to those plates with unconjugated antibody, and the plates were incubated for another 1 h at 37°C. Detection of reactivity was performed by using 100 μl/well of pNPP in diethanolamine (DEA) buffer (pNPP Microwell Substrate System, KPL) and the absorbance measured at intervals at 405 nm by an automatic ELISA Reader (BIORAD). All the assay conditions were previously set up for optimal concentrations through checkerboard and titration curves and all the reagents for antibody detection used in this assay were shown to be clear of non-specific reactions.

### Isolation of peripheral blood mononuclear cells and tissue cells for recall proliferation and cytokine culture

The mixture of blood/Alsever’s fluid was layered in two tubes containing 10 ml of density gradient solution (Ficoll-Paque™ Research Grade, Pharmacia, Uppsala, Sweden) and centrifuged at 2000 rpm for 20 min at room temperature. The peripheral blood mononuclear cells (PBMCs) were recovered from the gradient interphase of plasma and red blood cells, washed twice in RPMI 1640 medium containing 80 μg/ml gentamycin (Life Technologies, UK) by centrifuging 2000 rpm for10 min for the first time, and 1500 rpm for 10 min for the second time. The pellets were re-suspended in 10 ml of RPMI 1640 containing 10 μg/ml of Polymixin B, 10% FCS (GIBCO, Paisley, UK), 80 μg/ml gentamycin solution, 1% glutamine (200 mM), and 25 mM HEPES. For cells derived from spleen, mesenteric, and inguinal lymph nodes, the tissues were disrupted after having been pressed over a 40 μm cell strainer with a help of a syringe plunge. The cells were washed once in RPMI 1640 medium containing 80 μg/ml gentamycin (Life Technologies, UK) by centrifuging 2000 rpm for 10 min before the red blood cells were lysed for 10 min with lysing solution. After two more washes, the pellet was also re-suspended in 10 ml of RPMI 1640 containing 10 μg/ml of Polymixin B, 10% FCS (GIBCO, Paisley, UK), 80 μg/ml gentamycin solution, 1% glutamine (200 mM), and 25 mM HEPES. Viable cells were counted in a hemocytometer chamber using Trypan Blue dye exclusion (Sigma, St Louis, MO, USA). After cell count adjustment, 2 × 10^5^ cells were dispensed with 200 μl of RPMI 1640 containing 10% FCS (GIBCO, Paisley, UK), 80 μg/ml gentamycin solution, 1% glutamine (200 mM), and 25 mM Hepes into triplicate wells of a 96-well microtiter plates containing 20 μl of parasite antigen at 5 μg/ml (SWAP, SEA), 10 μg/ml (recombinant antigens), or 5 μg/ml of Con A. Plates were cultured at 37°C, 5% CO_2_ for 72 h, when 20 μl of 0.5 μCi of ^3^H thymidine was added per well. After incubation for another 18–20 h, the cells were harvested onto fiberglass filter papers before the thymidine incorporation was measured using 2 ml of scintillation fluid per filter in a Beta counter. Results were expressed as stimulation index (S.I.), the ratio of the mean counts per minute (cpm) of triplicate culture cells taken up in the presence of the antigen over those obtained with medium alone.

For cytokine cultures, 2 × 10^6^ cells were dispensed with 1000 μl of RPMI 1640 containing 10% FCS (GIBCO, Paisley, UK), 80 μg/ml gentamycin solution, 1% glutamine (200 mM), and 25 mM HEPES into each well of 48-well tissue culture plates containing 20 μl of parasite antigen at 25 μg/ml (Schistosome Soluble Worm Antigen Preparation, SWAP; Soluble Egg Antigen, SEA), 50 μg/ml (recombinant antigens), or 25 μg/ml of Con A. After 72 h of incubation at 37°C, 5% CO_2_, about 800 μl of supernatant/well were harvested and stored at −70°C until use. Freezing medium as 80%FCS/20% dimethyl sulfoxide (DMSO) was used in a drop wise manner in order to keep the remaining cells in cryo-freezing vials at 1 × 10^7^/ml concentration inside a liquid nitrogen tank.

### Analysis of soluble cytokines and chemokines secretion after antigenic stimulation

Harvested supernatants derived from PBMC cultures after stimulation with crude and recombinant antigens were assayed simultaneously using a 23-plex Non-Human Primate Cytokine/Chemokine Immunoassay Milliplex Kit (Millipore, Billerica, MA, USA) according to the manufacturer’s instructions, including quality controls. Briefly, 50 μl of standards or samples were incubated with multi-cytokine beads for 2 h in the dark, and following washes with a vacuum manifold (Millipore, Billerica, MA, USA) with biotinylated reporter for 1.5 h. The plates were then incubated with Streptavidin–Phycoerythrin for 30 min before the reaction was stopped for data collection in the Luminex 200 instrument using Luminex IS 2.3 software (Luminex Corporation, Austin, TX, USA) with a minimum of 50 beads per analyte. The resulting mean fluorescence intensity (MFI) was normalized and analyzed through the BeadView Multiplex Data Analysis Software version 1.0 (Millipore, Billerica, MA, USA), and expressed in pg/ml. The following cytokines and chemokines were assayed: IFN-γ, IL-12, IL-4, IL-6, IL-17A, CCL3 (MIP-1α), IL-5, IL-13, CCL2 (MCP-1), IL-1β, IL-2, IL-15, CCL4 (MIP-1β), TNF-α, IL-10, TGF-α, IL-1Ra, IL-8, GM-CSF, sCD40L, VEGF, G-CSF, IL-18.

### Statistical analysis

Statistical analyses were performed using GraphPad Prism (Prism 6 for Mac OS X) software. Parametric tests (ANOVA, student’s *t*-tests) were used after log-transformation with corrections for multiple analyses. Otherwise, non-parametric (Kruskal–Wallis, Mann–Whitney) tests were applied. Values were considered statistically significant when *p* < 0.05, and assigned *p = 0.01–0.05; **p = 0.001–0.01; and ***p = <0.001.

## Results

### Safety and toxicity (experiment 1)

As noted in Section “Immunizations and Challenge” and in Figure 1A, the Experiment 1 was for safety and toxicity evaluation after vaccination. All animals in the study were examined clinically throughout the study. The animals appeared healthy, eating normally without any physical signs of toxicity. The weight (Figure 1B) and temperature (Figure 1C) of each animal were normal throughout the study. In addition, as shown in Tables 1 and 2, the hematological values of the vaccinated animals remained within the normal limits throughout the observation period and there were no other clinical adverse signs. In addition, we examined the vaccine injection sites following immunization and did not observe any significant irritation at the vaccination site i.e., baboons did not experience any muscle transient induration, granulomas, abscesses, ulcers, cutaneous erythema, inguinal lymphadenopathy,or skin swelling at the vaccination sites (data not shown). In a similar manner, we examined baboons for adverse events due to vaccination in Experiment 2. There were none.

**Table 1:** **Summary of effects of vaccination in baboons (Experiment 1)^a^**.

Week		Time point	WBC (**×**10^3^)	Neu (%)	Eos (%)	Baso (%)	Lymp (%)	Mono (%)
CTSOD	0	PI	8.84 ± 2.53	61.2 ± 3.7	1 ± 0.71	0 ± 0	34.2 ± 0.96	2.6 ± 1.14
	2	Post-I	14.98 ± 5.09	59.2 ± 6.94	1.8 ± 1.30	0 ± 0	35 ± 9.08	3.8 ± 2.17
	4	Pre-boost 1	12.66 ± 2.0	58.8 ± 12.58	1.2 ± 1.30	0 ± 0	38.8 ± 11.99	1.2 ± 0.45
	6	Post-boost 1	11.48 ± 2.74	45.6 ± 10.78	1.2 ± 2.17	0 ± 0	49.8 ± 10.92	2.6 ± 1.34
	8	Pre-boost 2	10.68 ± 2.95	38.2 ± 15.75	2.2 ± 2.17	0 ± 0	59 ± 16.73	0.6 ± 0.89
	10	Post-boost 2	16.86 ± 4.77	41.6 ± 9.18	3.2 ± 1.64	0 ± 0	55 ± 8.34	0.2 ± 0.45
	12	Pre-boost 3	12.52 ± 4.04	50.4 ± 17.56	2.4 ± 1.67	0 ± 0	46.4 ± 16.52	0.6 ± 0.89
	15	Post-boost 3	9.08 ± 2.48	47 ± 14.02	1 ± 0	0.4 ± 0.55	51.2 ± 13.99	0.4 ± 0.89
GPX	0	PI	11.62 ± 3.71	57.8 ± 14.97	1 ± 1.0	0 ± 0	37 ± 15.44	2.8 ± 0.84
	2	Post-I	10.32 ± 2.66	46.5 ± 7.05	0.7 ± 0.5	0.25 ± 0.5	49.5 ± 7.72	2.75 ± 0.96
	4	Pre-boost 1	10.46 ± 2.37	44.4 ± 14.71	0.2 ± 0.45	0.2 ± 0.45	53 ± 14.18	2.2 ± 0.84
	6	Post-boost 1	12.92 ± 3.27	36.2 ± 4.44	0.6 ± 0.55	0 ± 0	59.8 ± 4.09	3.4 ± 1.52
	8	Pre-boost 2	12.56 ± 2.17	51.2 ± 10.92	0.8 ± 1.30	0 ± 0	44.6 ± 9.94	2.2 ± 0.84
	10	Post-boost 2	18.9 ± 5.03	30.8 ± 13.54	0.6 ± 0.89	0 ± 0	67.6 ± 11.93	1 ± 1.73
	12	Pre-boost 3	13.52 ± 2.53	40.4 ± 20.44	1 ± 0.71	0 ± 0	57.6 ± 20.01	1 ± 2.24
	15	Post-boost 3	15.54 ± 4.58	66.2 ± 5.17	1 ± 2.24	0 ± 0	32 ± 4.53	0.6 ± 0.55
Control		Normal range	6–25.3	21–81	0–6	0–1	17–83	0–5

^a^CTSOD *n* = 5, GPX *n* = 5, Control *n* = 10.

Values are expressed as mean average ± SD.

PI, pre-immune; Post-I, post-immune; WBC, total leukocyte count; Neu, neutrophils; Eos, eosinophils; Bas, basophils; Lymp, lymphocytes; Mono, monocytes.

**Table 2:** **Summary of effects of vaccination in baboons (Experiment 1)^a^**.

Week		Time point	RBC (**×**10^6^)	Hb (g/dl)	PVC (%)	MCV (fl)	MCH (pg)	MCHC(g/dl)
CTSOD	0	PI	4.302 ± 0.3	12.94 ± 0.93	38.8 ± 2.77	90.72 ± 11.5	30.26 ± 3.83	33.3 ± 0
	2	Post-I	4.06 ± 0.17	12.58 ± 0.36	33.5 ± 1.52	82.4 ± 1.12	30.98 ± 1.2	37.52 ± 1.48
	4	Pre-boost 1	4.626 ± 0.29	13.64 ± 0.15	37.86 ± 2.48	82.04 ± 1.66	29.54 ± 1.91	36 ± 2.29
	6	Post-boost 1	4.166 ± 0.23	13 ± 0.19	33.46 ± 2.69	80.3 ± 3.46	31.24 ± 1.81	39.02 ± 3.55
	8	Pre-boost 2	4.398 ± 0.35	13.56 ± 0.48	35.42 ± 4.46	80.34 ± 4.57	30.94 ± 2.58	38.7 ± 4.68
	10	Post-boost 2	4.634 ± 0.19	14.56 ± 0.21	39.04 ± 2.47	84.2 ± 2.37	31.4 ± 1.63	38.6 ± 2.58
	12	Pre-boost 3	4.248 ± 0.26	11.94 ± 1.15	35.42 ± 2.75	83.32 ± 3.24	27.92 ± 2.3	33.54 ± 3.04
	15	Post-boost 3	5.022 ± 0.19	12.34 ± 1.1	34.42 ± 1.20	68.5 ± 2.96	24.56 ± 2.12	35.88 ± 3.82
GPX	0	PI	4.264 ± 0.51	13.14 ± 0.76	40.34 ± 1.93	95.24 ± 9.91	31.28 ± 3.57	33.3 ± 0
	2	Post-I	4.59 ± 0.39	12.77 ± 0.55	38.25 ± 3.29	83.35 ± 0.17	27.9 ± 2.03	33.55 ± 2.6
	4	Pre-boost 1	4.79 ± 0.24	14.6 ± 0.86	39.92 ± 2.23	83.08 ± 2.98	31.08 ± 1.87	36.6 ± 2.95
	6	Post-boost 1	4.532 ± 0.43	13.78 ± 0.74	38.36 ± 6.42	88 ± 14.44	30.48 ± 1.46	35.58 ± 6.35
	8	Pre-boost 2	4.596 ± 0.26	14.64 ± 0.88	36.48 ± 2.06	79.36 ± 0.96	31.8 ± 0.23	40.08 ± 0.57
	10	Post-boost 2	4.348 ± 0.23	14.86 ± 0.96	36.42 ± 2.25	83.7 ± 1.46	34.14 ± 1.32	40.78 ± 1.99
	12	Pre-boost 3	4.554 ± 0.26	11.92 ± 0.34	39.82 ± 4.49	87.14 ± 4.91	26.18 ± 1.92	30.12 ± 3.99
	15	Post-boost 3	5.286 ± 0.52	13.58 ± 0.89	35.9 ± 3.23	68.1 ± 1.81	25.76 ± 2.17	37.96 ± 3.51
Control		Normal range	3.64–5.36	11.3–14.5	27.7–49.3	65.4–102	23.5–35.2	28.9–44.4

^a^CTSOD *n* = 5; GPX *n* = 5; Control *n* = 10.

Values are expressed as mean average ± SD.

PI, pre-immune; Post-I, post-immune; RBC, erythrocyte count; Hb, hemoglobin concentration; PVC, packed cell volume; MCV, mean corpuscular volume; MCH, mean corpuscular hemoglobin; MCHC, mean corpuscular hemoglobin concentration.

### Diarrhea, duration of dysentery, and egg excretion (experiment 2)

The safety of our vaccine candidates encouraged us to proceed with Experiment 2 (outlined in Figure 2A), where groups of five baboons each were vaccinated with CTSOD and GPX, as well as ECSOD, and challenged with 600 *S. mansoni* cercariae. Weekly clinical analysis demonstrated that all three vaccinated groups had higher percentage of animals without diarrhea (CTSOD = 60%, ECSOD = 60%, and GPX = 40%) upon the onset of egg deposition when compared to only 20% in the Control group (Figure 2B). In addition, animals vaccinated with antioxidants showed fewer mean days of dysentery compared to controls (Figure 2C), where CTSOD group presented with a mean reduction of 75% (ranging from 100 to 17%), ECSOD with 63% (ranging from 100 to 0%), and GPX with 25% (ranging from 100 to 0%).

**Figure 3:**
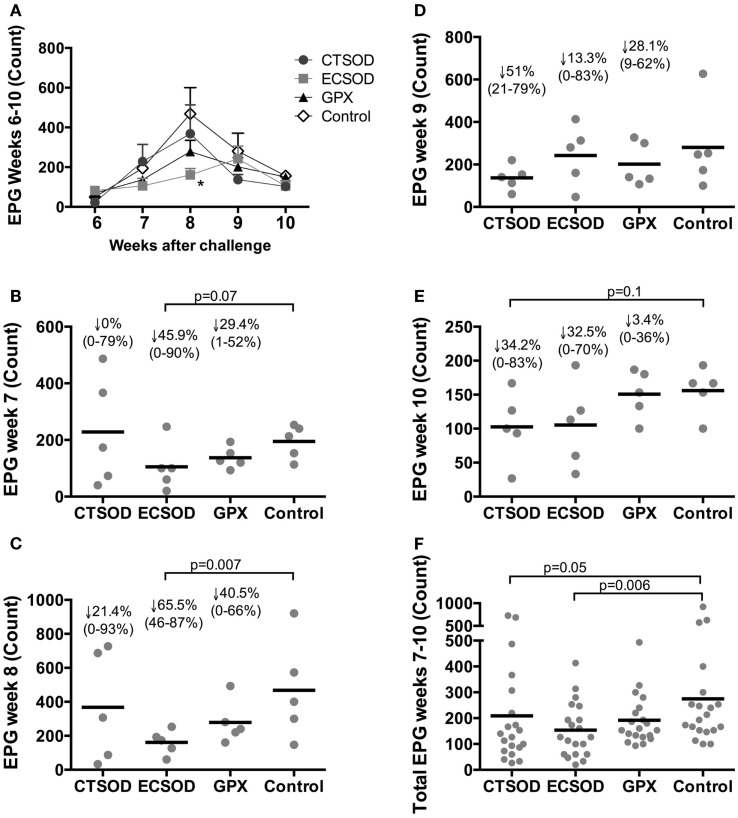
**Dynamics of weekly egg excretion per gram of feces (EPG) from Experiment 2**. Assessment of eggs per gram of feces (EPG) from vaccinated and control baboons (*n* = 5 animals per group) commencing 6 weeks after *S. mansoni* cercariae challenge: weekly overview **(A)**; week 7 **(B)**; week 8 **(C)**; week 9 **(D)**; week 10 **(E)**; and the total EPG excreted over 5 weeks period **(F)**. The statistical *p*-value was expressed in comparison to the mean average of the Control group. Results were log-transformed and corrected for multiple analyses when applying ANOVA and student’s *t*-tests. Values were considered statistically significant when *p* < 0.05.

The weekly assessment of the EPG commencing 6 weeks after challenge showed that vaccinated groups had an overall reduction of eggs when compared to the Control group (Figures 3A–F). This reduction was statistically significant within ECSOD at week 8 (Figure 3C) when compared to the unvaccinated Control group. When the total EPG over a 5-week period was analyzed, all vaccinated groups showed reduction of excreted eggs, specially ECSOD vaccinated animals who showed statistically significant reduction when compared to the Control (Figure 3F).

### Tissue eggs from liver, small gut, and large gut (experiment 2)

**Figure 4:**
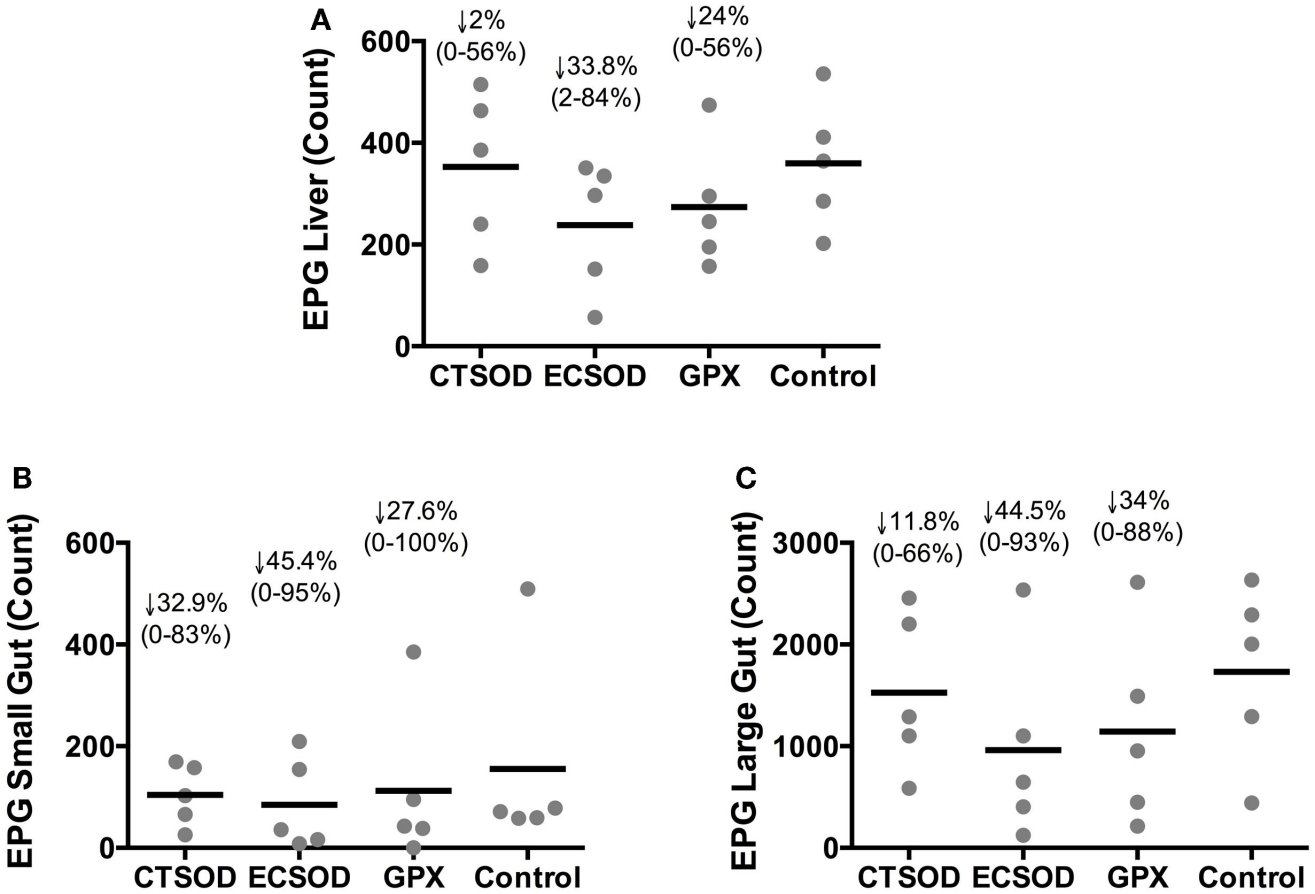
**Distribution of eggs recovered in tissues from Experiment 2**. Liver **(A)**, small gut **(B)**, and large gut **(C)** eggs after KOH digestion from vaccinated and control baboons (*n* = 5 animals per group) 0 weeks after challenge with *S. mansoni* cercariae (perfusion time point). Individual values (sphere); mean average (dash). The percentage of reduction (represented by the arrow), the range (minimal–maximal, represented between brackets), and the statistical *p*-value were expressed in comparison to the mean average of the Control group. Non-parametric (Kruskal–Wallis, Mann–Whitney) tests were applied. Values were considered statistically significant when *p* < 0.05.

The distribution of eggs recovered in harvested organs after KOH digestion from vaccinated and control baboons 10 weeks after challenge at perfusion time point is shown in Figure 4. There was an overall reduction of eggs in all vaccinated groups in the liver, small gut, and large gut when compared to the Control group. The egg reduction in the liver (Figure 4A) ranged from 56 to 0% for CTSOD, 84 to 3% for ECSOD, and 56 to 0% for GPX, while in the small gut (Figure 4B), the reduction in CTSOD group ranged from 64 to 0%, ECSOD ranged from 88 to 0%, and GPX ranged from 100 to 0%. When the large gut was evaluated (Figure 4C), egg reduction ranged from 70 to 0% for CTSOD, 93 to 0% for ECSOD, and 89 to 0% for GPX, when compared to the mean average of Control group.

### Worm burden/percentage of protection (experiment 2)

**Figure 5:**
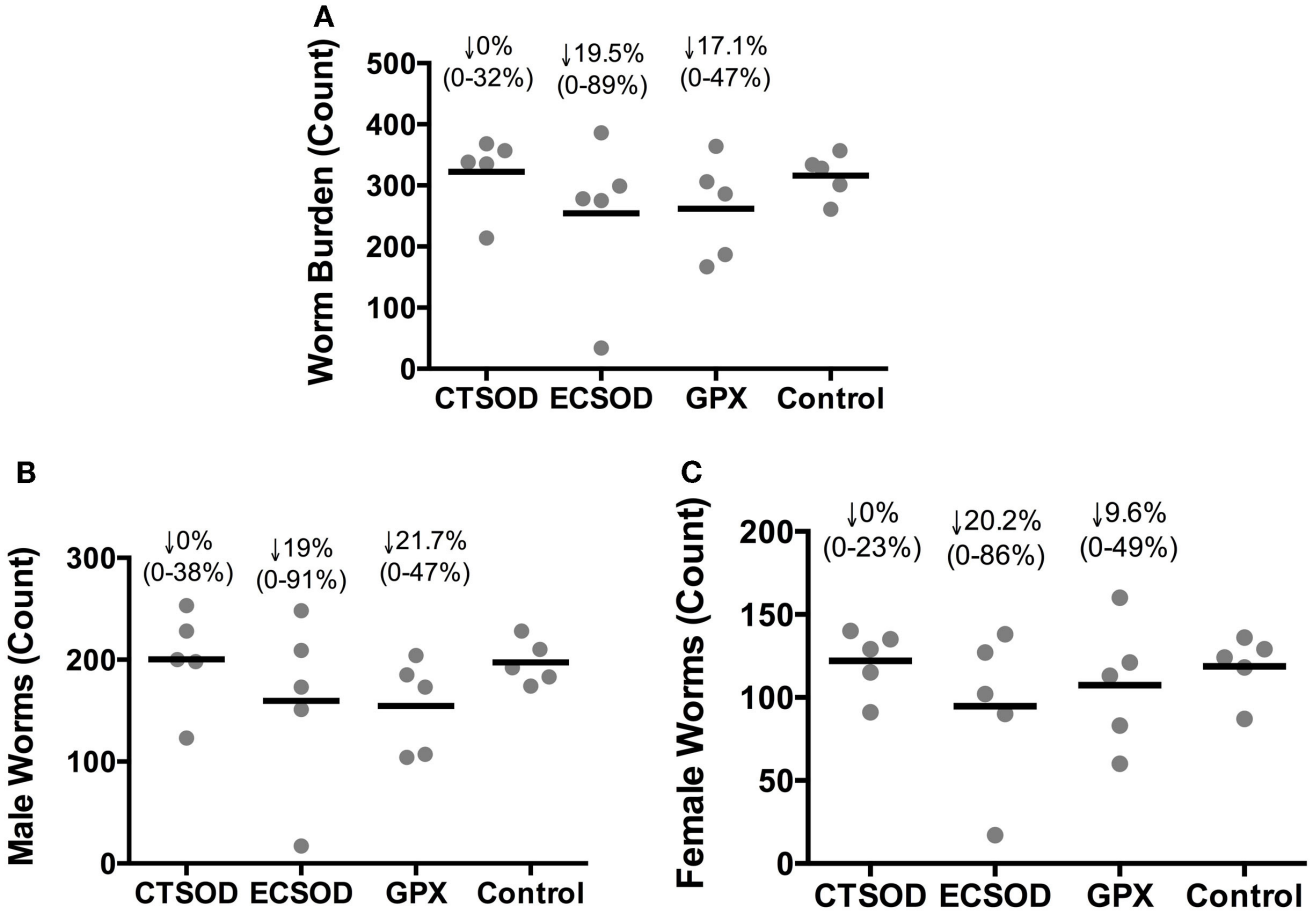
**Distribution of worms recovered (worm burden) from Experiment 2**. Total worm burden **(A)**, male **(B)** and female **(C)** worm count. Worms from vaccinated and control baboons (*n* = 5 animals per group) 10 weeks after challenge with *S. mansoni* cercariae (perfusion time point). Individual values (sphere); mean average (dash). The percentage of reduction (represented by the arrow), the range (minimal–maximal, represented between brackets), and the statistical *p*-value were expressed in comparison to the mean average of the Control group. Non-parametric (Kruskal–Wallis, Mann–Whitney) tests were applied. Values were considered statistically significant when *p* < 0.05.

When the distribution of worms (Figure 5A), males (Figure 5B), and females (Figure 5C) recovered from outbred vaccinated and control baboons 10 weeks after challenge with *S. mansoni* cercariae was evaluated at the perfusion time point, ECSOD vaccinated group showed a reduction of total worms that ranged from 89 to 0% (mean protection 19.5%), while GPX group showed a reduction that ranged from 47 to 0% (mean protection 17.1%) of worms when compared to the Control group (Figure 5A). The same pattern was observed when worms were stratified into males (Figure 5B) and females (Figure 5C). The CTSOD group failed to show a mean reduction in worms, although reduction in two vaccinated baboons (out of five) is visible.

### Antibody response (experiment 2)

**Figure 6:**
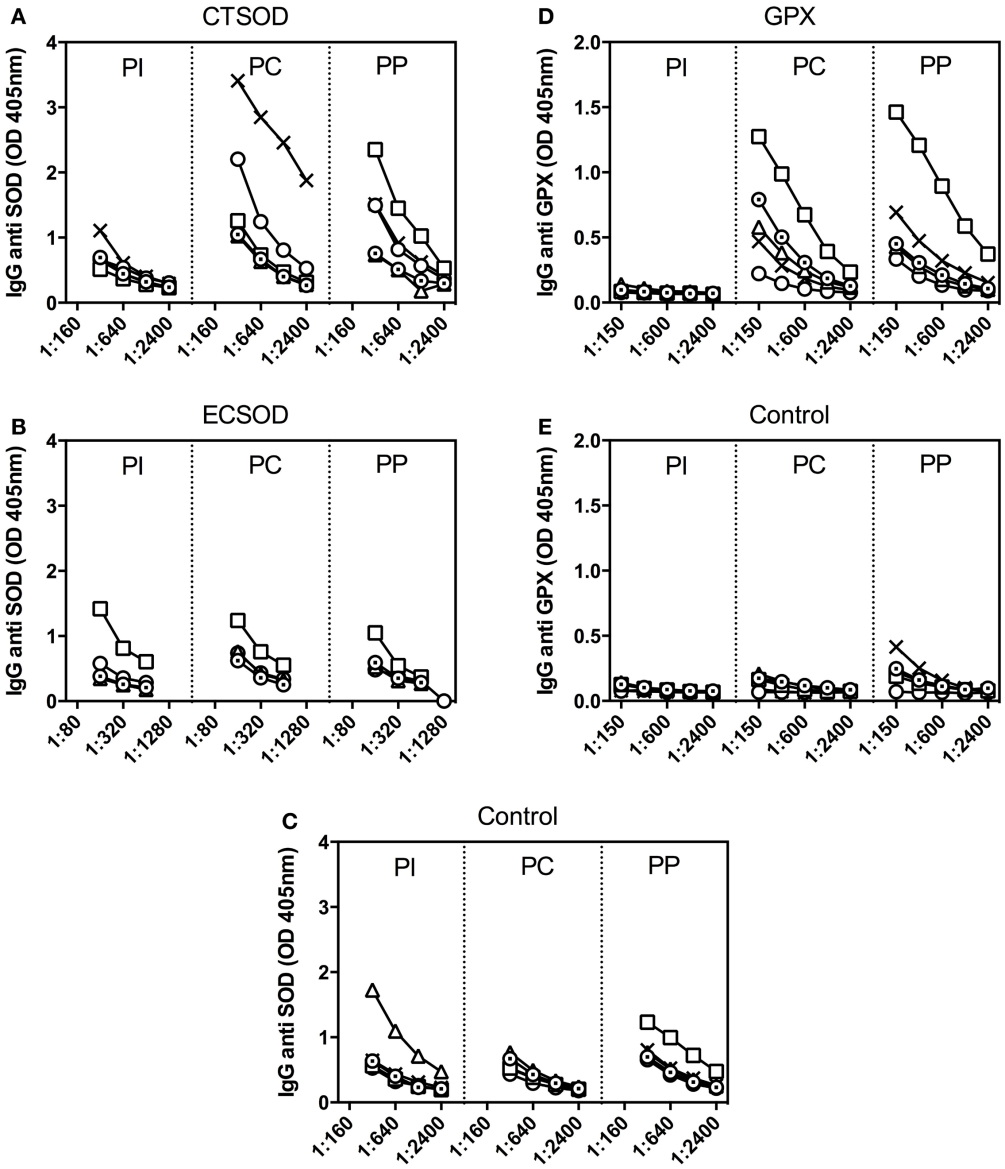
**Antibody response from Experiment 2**.Titration of baboon IgG antibodies against recombinant SOD **(A–C)** and GPX **(D–E)** from CTSOD **(A)**, ECSOD **(B)**, GPX **(D)**, and Control **(C,E)** groups (*n* = 5 animals per group) before (PI), after immunizations (pre-challenge, PC), and before perfusion (pre-perfusion, PP).

Titration of baboon sera against recombinant antigens showed that levels of specific IgG antibodies were stimulated after immunizations (PC) with CTSOD (Figure 6A) as well as with GPX (Figure 6D), and to a lesser degree with ECSOD (Figure 6B), when compared to control sera (Figures 6C,E). Antibody levels continued to be elevated 10 weeks after challenge infection (PP) for CTSOD and GPX groups.

### CMI proliferation/recall responses (experiment 2)

**Figure 7:**
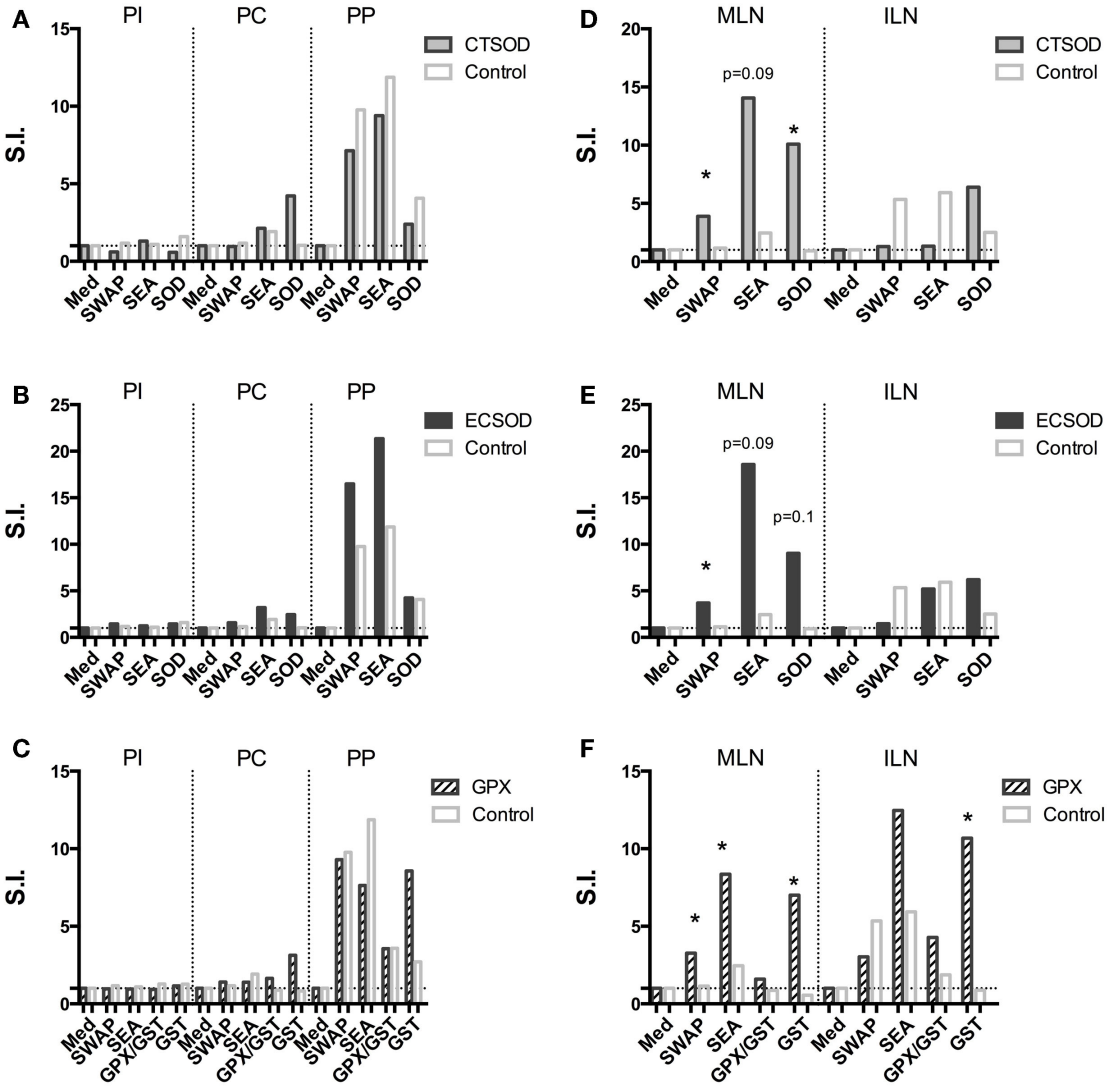
**Recall proliferation assays from Experiment 2**. PBMC **(A–C)** and mesenteric (MLN) and inguinal (ILN) lymph nodes **(D–F)** against crude and recombinant antigens from CTSOD **(A,D)**, ECSOD **(B,E)**, GPX **(C,F)**, and Control groups (*n* = 5 animals per group) before (PI), after immunizations (pre-challenge, PC), and after challenge with *S. mansoni* at the perfusion time point (PP). Results were expressed as Stimulation Index (S.I.), the ratio of the mean counts per minute (cpm) of triplicate culture cells taken up in the presence of the antigen over those obtained with medium alone. Non-parametric (Kruskal–Wallis, Mann–Whitney) tests were applied. Values were considered statistically significant when *p* < 0.05.

Recall proliferation assays from PBMCs (Figures 7A–C) were performed against crude and recombinant antigens before (pre-immune, PI), after immunizations (pre-challenge, PC), and after challenge with *S. mansoni* at the perfusion time point (PP). The recall proliferation assays for mesenteric (MLN) and inguinal (ILN) lymph nodes (Figures 7D–E) were performed only at the time of the perfusion (PP). All results were expressed as Stimulation Index (S.I.): the ratio of the mean cpm of triplicate culture cells taken up in the presence of the antigen over those obtained with medium alone. PBMC (Figures 7A,B) and MLN cells (Figures 7D,E) from both CTSOD and ECSOD groups proliferated in response to recombinant CTSOD antigens when compared with Control. Similarly, PBMC (Figure 7C), mesenteric, and inguinal node cells (Figure 7F) from GPX vaccinated animals proliferated in response to recombinant antigens in comparison with Control group. PBMC and MLN from all groups responded to SEA and SWAP after challenge with *S. mansoni* cercariae (Figures 7D–F).

### Secretion of cytokines and chemokines upon stimulation (experiment 2)

**Figure 8:**
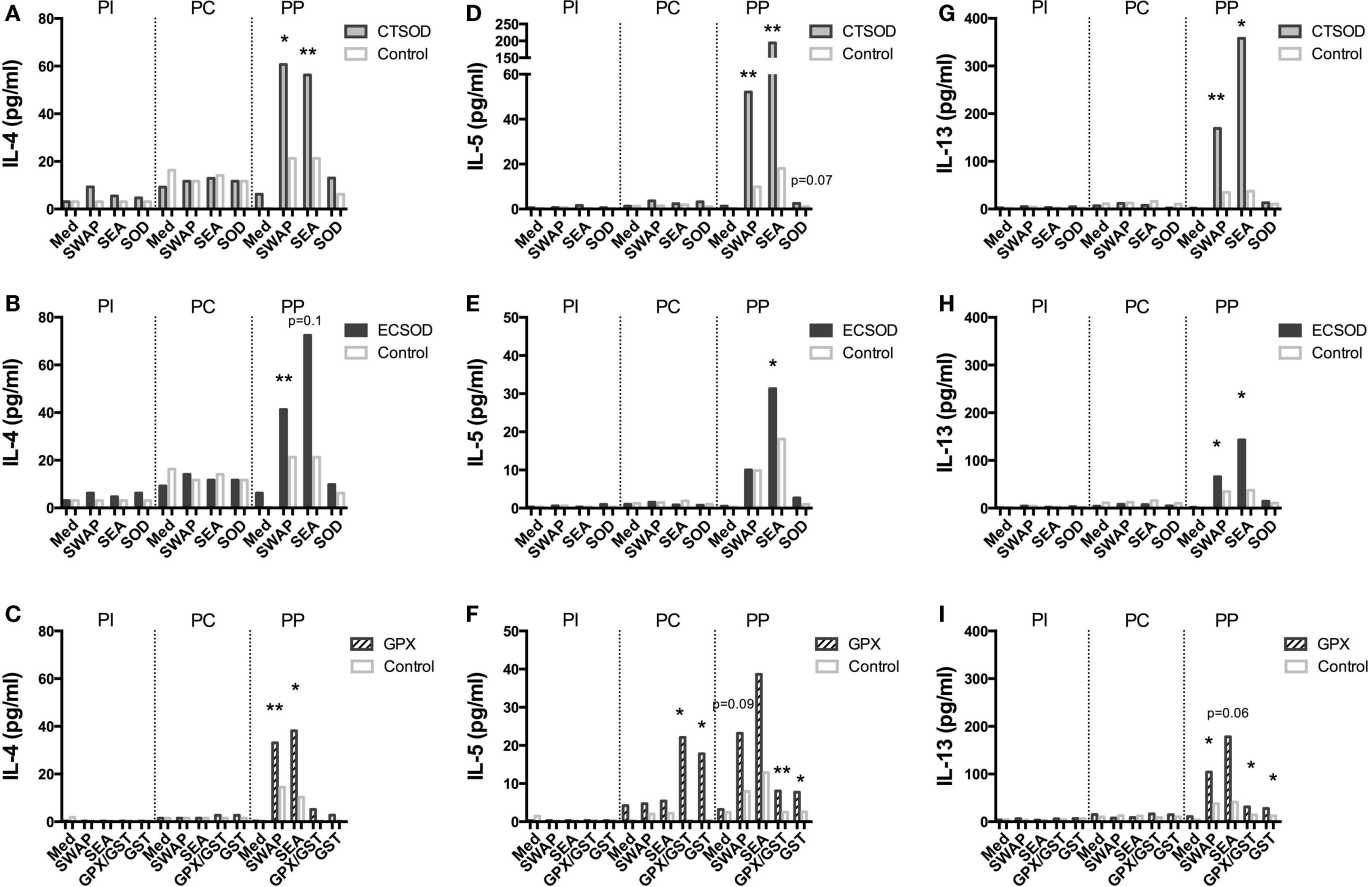
**Secretion of cytokines in supernatants from Experiment 2**. PBMCs from CTSOD **(A,D,G)**, ECSOD **(B,E,H)**, and GPX **(C,F,I)** groups (*n* = 5 animals per group) were stimulated 72 h with crude and recombinant antigens and secreted IL-4, IL-5, and IL-13 levels determined, before vaccination (PI), before challenge (PC), and at perfusion (PP). Values were expressed as median of cytokine secretion in five individual supernatants in relation to the Control baboons. Non-parametric (Kruskal–Wallis, Mann–Whitney) tests were applied. Values were considered statistically significant when *p* < 0.05, and assigned *p = 0.01–0.05; **p = 0.001–0.01; and ***p = <0.001.

**Figure 9:**
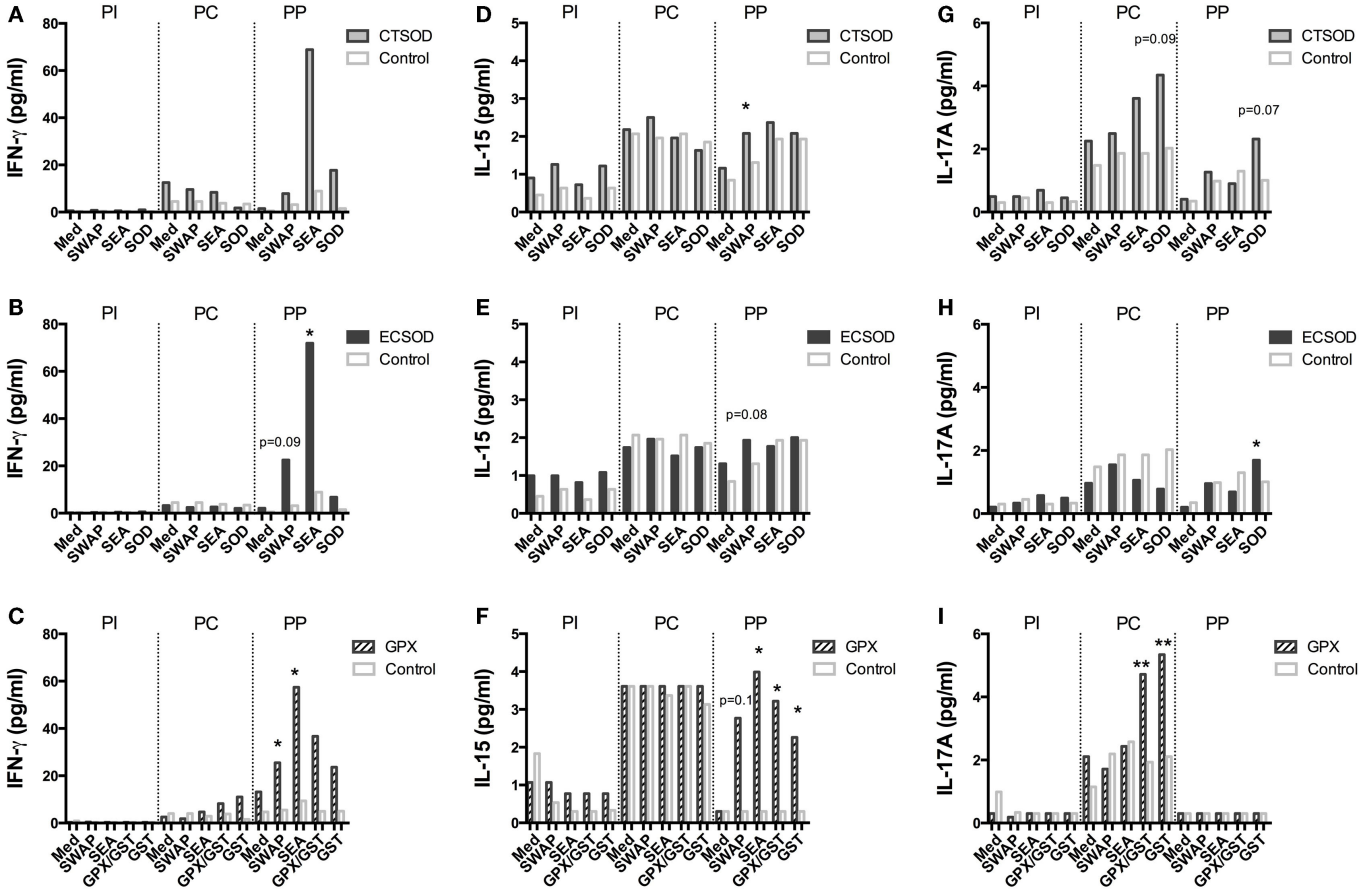
**Secretion of cytokines in supernatants from Experiment 2**. PBMCs from CTSOD **(A,D,G)**, ECSOD **(B,E,H)**, and GPX **(C,F,I)** groups (*n* = 5 animals per group) were stimulated 72 h with crude and recombinant antigens and secreted IFN-γ, IL-15, and IL-17A levels determined, before vaccination (PI), before challenge (PC), and at perfusion (PP). Values were expressed as median of cytokine secretion in five individual supernatants in relation to the Control baboons. Non-parametric (Kruskal–Wallis, Mann–Whitney) tests were applied. Values were considered statistically significant when *p* < 0.05, and assigned *p = 0.01–0.05; **p = 0.001–0.01; and ***p = <0.001.

**Figure 10:**
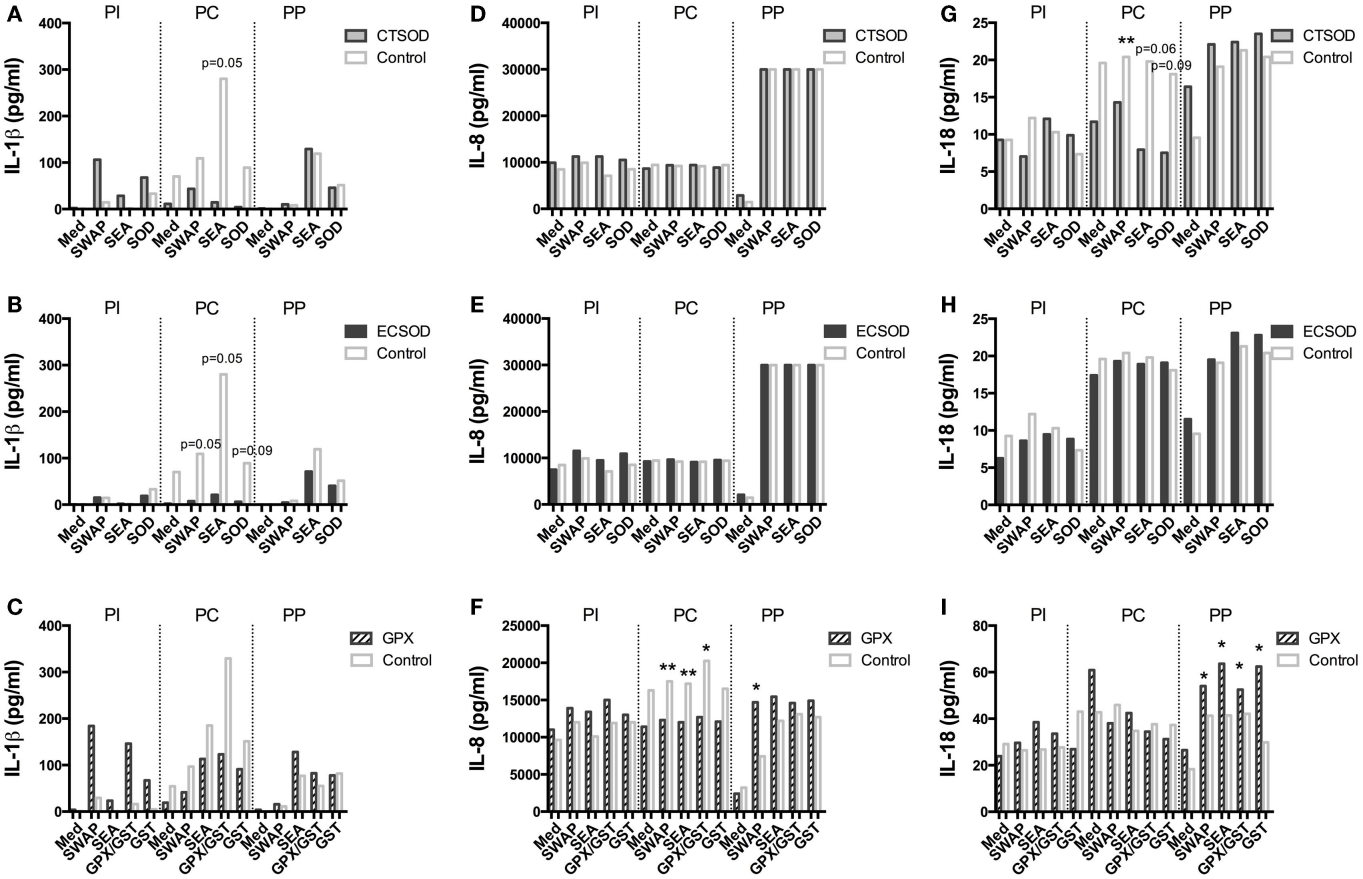
**Secretion of cytokines in supernatants from Experiment 2**. PBMCs from CTSOD **(A,D,G)**, ECSOD **(B,E,H)**, and GPX **(C,F,I)** groups (*n* = 5 animals per group) were stimulated 72 h with crude and recombinant antigens and secreted IL-1β, IL-8, and IL-18 levels determined, before vaccination (PI), before challenge (PC), and at perfusion (PP). Values were expressed as median of cytokine secretion in five individual supernatants in relation to the Control baboons. Non-parametric (Kruskal–Wallis, Mann–Whitney) tests were applied. Values were considered statistically significant when *p* < 0.05, and assigned *p = 0.01–0.05; **p = 0.001–0.01; and ***p = <0.001.

**Figure 11:**
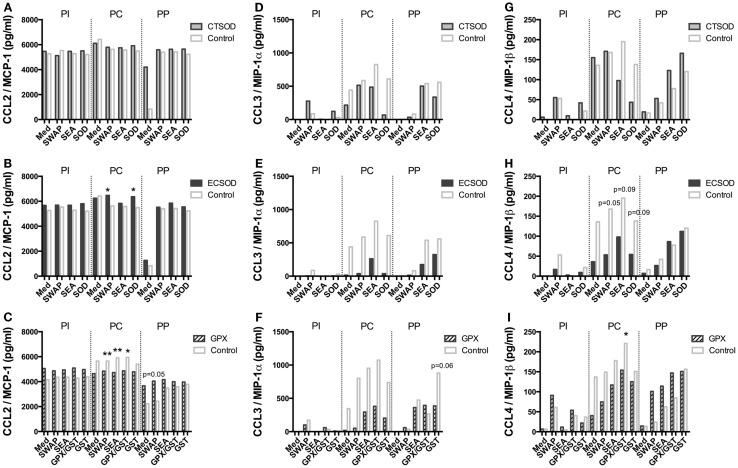
**Secretion of chemokines in supernatants from Experiment 2**. PBMCs from CTSOD **(A,D,G)**, ECSOD **(B,E,H)**, and GPX **(C,F,I)** groups (*n* = 5 animals per group) were stimulated 72 h with crude and recombinant antigens and secreted CCL2 (MCP-1), CCL3 (MIP-1α), and CCL4 (MIP-1β) levels determined, before vaccination (PI), before challenge (PC), and at perfusion (PP). Values were expressed as median of cytokine secretion in five individual supernatants in relation to the Control baboons. Non-parametric (Kruskal–Wallis, Mann–Whitney) tests were applied. Values were considered statistically significant when *p* < 0.05, and assigned *p = 0.01–0.05; **p = 0.001–0.01; and ***p = <0.001.

A panel of 23 multiplexed IFN-γ, IL-12, IL-4, IL-6, IL-17A, CCL3 (MIP-1α), IL-5, IL-13, CCL2 (MCP-1), IL-1β, IL-2, IL-15, CCL4 (MIP-1β), TNF-α, IL-10, TGF-α, IL-1Ra, IL-8, GM-CSF, sCD40L, VEGF, G-CSF, IL-18 cytokines and chemokines (Figures 8–11; Figures S1 and S2 in Supplementary Material) was used to investigate secretion from individual supernatants derived from PBMCs from CTSOD (A, D, G), ECSOD (B, E, H), and GPX (C, F, I) groups after 72 h stimulation with crude and recombinant antigens at all time points: before vaccination (PI), before challenge (PC), and at perfusion (PP). Values were expressed as median of cytokine secretion from five individual supernatants.

In general, vaccination with ECSOD and CTSOD induced variable levels of cytokines before challenge (PC) upon stimulation with recombinant antigens when compared to the baseline levels (PI), although these levels were somewhat discrete. However, vaccination with GPX induced higher levels of IL-5 (Figure 8F) and IL-17A (Figure 9I) cytokines, and reduction of IL-8 (Figure 10F) and chemokines such as CCL2, CCL3, and CCL4 (Figures 11C,F,I) when compared to Control. CTSOD vaccination induced increase in the IL-17A levels (Figure 9G), while a reduction of IL-1β, IL-18 (Figures 10A,B,G,H) cytokines, and CCL3 and CCL4 (Figures 11C,I) chemokines in CTSOD and ECSOD groups was also observed, when compared to Control.

After challenge, several cytokine and chemokine levels increased in the vaccinated groups after the natural boost provided from the infection with *S. mansoni* (Figures 8–11). For instance, high levels of IL-4, IL-5, IL-13 (Figures 8A–I), IFN-γ (Figures 9A–C) cytokines were stimulated after challenge (PP) against SWAP and SEA in the CTSOD, ECSOD, and GPX groups when compared to (PC) levels, and this response was higher in the vaccinated groups when compared to the control group. The levels of IL-15 (Figures 9D–F) were also increased, especially in the GPX group, before perfusion (PP) when compared to control.

## Discussion

As mentioned previously, our laboratory has demonstrated that vaccination of inbred mice with naked DNA constructs containing Cu/Zn cytosolic superoxide dismutase (CT-SOD), signal-peptide containing SOD (EC-SOD), or glutathione peroxidase (GPX) derived from *S. mansoni* showed significant levels of protection compared to a control group (29). Screening of candidate antigens in mice is an important first step in vaccine development but it is unclear whether protective efficacy can be translated directly to humans (38). Therefore, our murine results encouraged us to investigate if immunization of non-human primates with these antioxidants would stimulate an immune response that could be correlated with protection as a prelude study for human trials. Baboons are an excellent model for schistosomiasis, as they are similar to humans in ontogeny, immune response (including human-like IgG subclasses), reproductive physiology, etc., and develop a human-like acute disease after natural or experimental exposure to *S. mansoni* (38–40). In addition, since issues of vaccine safety are also difficult to address in mice, another advantage of testing schistosome vaccines in baboons is the opportunity to address many of the deficiencies of mouse studies (38–41). We have also previously used baboons to successfully investigate the immune mechanisms associated with other schistosome vaccine candidates (42). Overall, our data indicate that no adverse reactions or abnormal animal demeanor occurred to the immunization of baboons with antioxidants, as our behavioral, clinical, and hematological evaluations of the vaccinated animals remained within the normal limits throughout the observation period for both experiments. Thus the vaccines are safe and well tolerated.

The next step was to check the antigenicity of the antioxidants after vaccination with baboons, as the dose and nature of the antigen as well as the route or kind of adjuvant used can ultimately dictate the outcome of an immunization, among other factors. Plasmid/naked DNA vaccines are less toxic, but also less immunogenic, therefore potent and safe adjuvants that can be used as vaccine delivery systems and as immunostimulatory adjuvants are necessary (43). Biodegradable and biocompatible polyester polymers such as PLA to encapsulate antigens (44–46) have been successfully used in applications ranging from cancer therapy to infectious diseases (47–50). The enhanced adjuvant effect of such microparticles appears to be a consequence of efficient and controlled delivery of the adsorbed proteins into dendritic cells and macrophages at the injection site and local lymph nodes (51, 52). In the murine model, we showed that along with DNA immunization, one single dose of SmCT-SOD proteins encapsulated in PLA microspheres, for instance, was able to induce high titers of specific antibodies in immunized mice (32). Therefore, in our baboon studies, we evaluated if the immunization protocol of priming with naked DNA and boosting with the respective antioxidant proteins encapsulated in PLA microspheres could meet or even enhance the immunogenicity and protection levels achieved by prime-boost immunization with DNA only. All three antioxidant vaccines were immunogenic to different degrees, stimulating both humoral and cellular responses (including cytokines and chemokines). As observed previously by us and by other studies, the specific antibody and cellular responses primed by DNA vaccination were boosted by infection. This natural boosting is believed to be beneficial in endemic areas where individuals are continuously exposed to the parasite (31, 53, 54).

Numerous studies on individuals in endemic areas for *Schistosoma* showed that parasite-specific humoral and cellular responses vary in their correlation with the development of resistance and/or susceptibility/morbidity to infection/re-infection (55–57). And an increasing body of evidence indicates that a balance between innate cells and CD4+ T helper (Th) cells Th1, Th2, Th17, and Treg responses (which cross-regulate one another during infection) rather than a polarizing effect (e.g., Th1 vs Th2) is likely beneficial in the development of protection against *S. mansoni* infections, both in humans and experimental models (58–61). Moreover, it is becoming evident that the interaction between these responses rather than just the levels of individual cytokines alone may influence outcomes such as resistance to reinfection (62, 63).

In our study, a mixed cytokine/chemokine response was observed, where the *S. mansoni* infection stimulated a Th2 response in all groups as well as an inflammatory profile that differed from controls. Although it was clear that vaccination with ECSOD, CTSOD, and GPX stimulated different responses when compared to the control group. The specific role of antibodies or cellular responses and the development of protection against challenge with *S. mansoni* is still unknown, and currently under investigation. As shown by Mola et al. (64), the levels of most cytokines produced by PBMC or antibodies vary depending on whether the baboons experienced a primary infection compared to re-infection with *S. mansoni*. Although the young baboons used in this study were caught in the wild from non-endemic areas and were quarantined and checked for the presence of current schistosomiasis infection (and other infections), we cannot rule out the possibility that some of these animals were infected prior to the beginning of our study, therefore influencing the cytokine responses. However, this in fact would most likely resemble the situation in endemic areas, where people in endemic areas would be exposed to one or multiple infections or none at all before vaccination with antioxidants, and in many instances, such exposure to worm antigens in humans would have occurred very early in life during childhood or even *in utero* (65, 66), which makes outbred baboons ideally suited for such studies.

In schistosomiasis, pathogenesis is mainly caused by the immunological reaction of the host to the eggs (and their secretions) released by adult worm pairs that inhabit the portal circulation. Consequently, the severity of the disease is directly related to the worm burden and the inflammatory response to deposited eggs (67). For instance, as recently reviewed (68), the chemokines CCL2, CCL3, and CCL4 correlate with the severity of *S. mansoni*, where CCL3 is related to the recruitment of eosinophils and induction of granulomatous pathology (69), while CCL2 is associated with glomerulopathy (70). A more “traditional” vaccine concept would target a reduction of the worm number or even prevent infection. However, since the released eggs are responsible for both transmission and pathology, a vaccine targeted at parasite fecundity and egg viability has become relevant (71, 72). The vaccination of baboons with antioxidants in the present study stimulated a partial reduction in the worm burden, which was in sharp contrast with our previous studies in mice (29). However, vaccination with antioxidants, especially ECSOD, promoted a strong anti-fecundity and anti-pathology effect, by means of an overall reduction of eggs in all vaccinated groups in the feces, liver, small gut, and large gut, as well as reduction of diarrhea (dysentery) when compared to the Control group. One of the hallmarks of pathology, diarrhea normally occurs during moderate and heavy *S. mansoni* infections due to eggs released by the worms, which in turn induce an immunological response leading to pathology in the intestine wall (67). In our study, diarrhea was reduced in all vaccinated groups, in particular in the CTSOD and ECSOD groups. Other studies had reported both immune responses and anti-fecundity effects against female worms with glutathione-S-transferase, SmGST (73, 74), as well as the large subunit of calpain, Sm-p80 (75). Interestingly, the significant reduction in fecundity in this study was in spite of the small reduction in worm burden. The mechanism of the anti-fecundity effect by antioxidants or any other vaccine candidate, which seems to be independent of worm burden, remains to be determined.

Taken together, our results challenge the common concept for markers and correlates of protection, since in despite of the lower than 40% worm reduction, as set by WHO, the high anti-fecundity/anti-pathology effect observed by means of reduction of eggs and overall pathology indicates that these antioxidants vaccines could prevent intestinal pathology and therefore warrants further investigation. In addition, the use of outbred juvenile baboons more accurately reflects the human situation as regards response to vaccination and subsequent challenge infection. Experiments with different vaccine regimens (including multivalent immunizations), delivery modes, and adjuvants in the baboon model are planned for the near future.

## Conflict of Interest Statement

The authors declare that the research was conducted in the absence of any commercial or financial relationships that could be construed as a potential conflict of interest.

## Supplementary Material

The Supplementary Material for this article can be found online at http://journal.frontiersin.org/article/10.3389/fimmu.2015.00273/abstract
