# Supplementary material for: Protective Potential of Antioxidant Enzymes as Vaccines for Schistosomiasis in a Non-Human Primate Model
Source: Front Immunol. 2015 Jun 2;6:273. doi: 10.3389/fimmu.2015.00273 (PMC4451692; doi:10.3389/fimmu.2015.00273)
Supplement: Supplementary file 2 [file image_2.pdf]

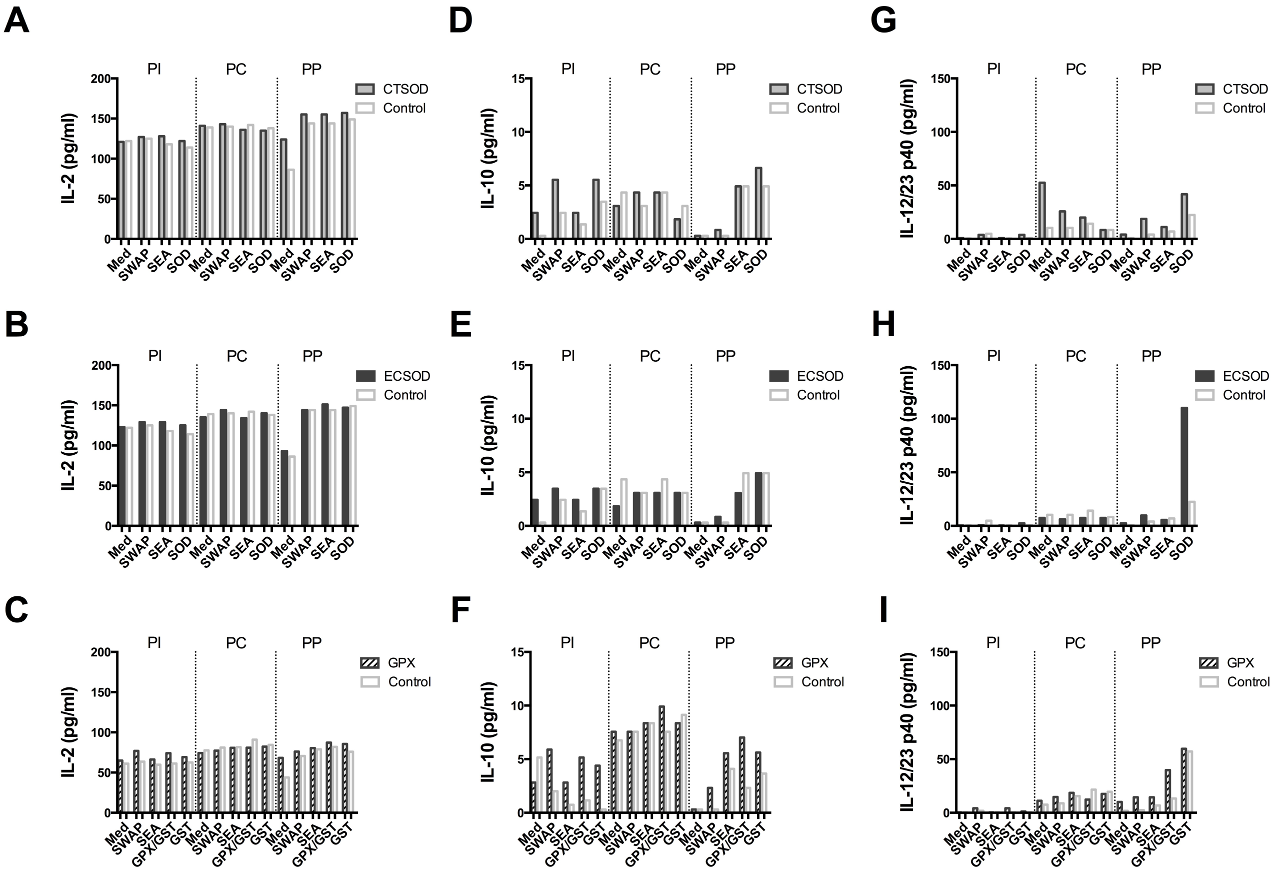

Supplemental Figure 2 | Secretion of cytokines in supernatants from Experiment 2. PBMCs from CTSOD (A, D, G), ECSOD (B, E, H) and GPX (C, F, I) groups (n=5 animals per group) were stimulated 72h with crude and recombinant antigens and secreted IL-2, IL-10 and IL-12/23 p40 levels determined, before vaccination (PI), before challenge (PC) and at perfusion (PP). Values were expressed as median of cytokine secretion in 5 individual supernatants in relation to the Control baboons. Non-parametric (Kruskal-Wallis, Mann-Whitney) tests were applied. Values were considered statistically significant when $p < 0.05$, and assigned * p =0.01 to 0.05; ** p =0.001 to 0.01; and *** p =< 0.001.
